# Chronic kidney disease: a review of proteomic and metabolomic approaches to membranous glomerulonephritis, focal segmental glomerulosclerosis, and IgA nephropathy biomarkers

**DOI:** 10.1186/s12953-019-0155-y

**Published:** 2019-12-20

**Authors:** Amir Taherkhani, Reyhaneh Farrokhi Yekta, Maede Mohseni, Massoud Saidijam, Afsaneh Arefi Oskouie

**Affiliations:** 1 Research Center for Molecular Medicine, Hamadan University of Medical Sciences, Hamadan, Iran; 2 Proteomics Research Center, Shahid Beheshti University of Medical Sciences, Tehran, Iran; 3 Urology and Nephrology Research Center, Hamadan University of Medical Sciences, Hamadan, Iran; 4 Department of Basic Sciences, Faculty of Paramedical Sciences, Shahid Beheshti University of Medical Sciences, Tehran, Iran

**Keywords:** Biomarker, Focal segmental glomerulonephritis, IgA nephropathy, Membranous glomerulonephritis, Proteomics

## Abstract

Chronic Kidney Disease (CKD) is a global health problem annually affecting millions of people around the world. It is a comprehensive syndrome, and various factors may contribute to its occurrence. In this study, it was attempted to provide an accurate definition of chronic kidney disease; followed by focusing and discussing on molecular pathogenesis, novel diagnosis approaches based on biomarkers, recent effective antigens and new therapeutic procedures related to high-risk chronic kidney disease such as membranous glomerulonephritis, focal segmental glomerulosclerosis, and IgA nephropathy, which may lead to end-stage renal diseases. Additionally, a considerable number of metabolites and proteins that have previously been discovered and recommended as potential biomarkers of various CKD_s_ using ‘-omics-’ technologies, proteomics, and metabolomics were reviewed.

## Background

Abnormal structure or function of the kidney, which can be accompanied by reduced Glomerular Filtration Rate (GFR), is known as Chronic Kidney Disease (CKD) [1]. Each year, CKD affects millions of people from all backgrounds and nations [2]. One of the problems associated with CKD is that, there are no early clinical symptoms to be used for diagnosis before the kidney enters an irreversible functional stage [3]. CKD also increases risk of cardiovascular diseases, hospitalization, and mortality [2, 4]. However, people with CKD have at least one of the following clinical signs: abnormal kidney structure (after imaging), reduced GFR, proteinuria, (or albuminuria), cellular deposits in their urine, or hematuria. The GFR is known as the best measure of renal function. It varies according to age, sex, and body size. The GFR is about 120–130 ml/min per 1.73 m^2^ in the youth. After 30 years of age, it annually decreases by an average of 1 ml/min per 1.73 m^2^ of the body’s surface. When the GFR reaches 90 ml/min, the first stage of CKD begins, and, when it reaches below 15 ml/min, it will cause renal kidney failure, also called as End-Stage Renal Disease (ESRD); in such a case, dialysis is inevitable. Creatinine clearance is used to estimate the GFR, but it is not a sensitive measure in early stages of CKD. When the GFR reduces to 33%, the serum creatinine may be enhanced from 0.8 to 1.2 mg/dl [1]. Thus, in the current review, it was attempted to explain pathogenesis, symptoms, and biomarkers of some of the most high-risk CKD_s_. A remarkable number of metabolites and proteins have previously been discovered and recommended as potential biomarkers of various CKD_s_ using ‘-omics-’ technologies [5–10]. These technologies include high-throughput methods, which might concurrently detect a large number of bio-molecules such as genes, transcriptomes, proteins, and metabolites in complex bio-samples including serum, plasma, tissue, and urine [8]. Proteomics and metabolomics are referred to the study of complete profile of proteins and metabolites respectively, at the time of sampling [11, 12]. Both have some advantages compared to the other fields of ‘-omics-’ (e.g., genomics and transcriptomics). Unlike genome, the proteome is dynamic, and is influenced by different circumstances such as occurrence of a disease. On the other hand, metabolome is downstream product of proteome, and is closer to the phenotype of a biological system in comparison with genome, transcriptome, and proteome [12, 13]. A number of technologies are utilized for biomarker discovery in the field of proteomics, mainly including 2-Dimensional gel Electrophoresis (2DE), 2D-differential gel electrophoresis, microarrays, mass spectrometry-based approaches such as Matrix-Assisted Laser Desorption/Ionization-*Mass* Spectrometry (MALDI-MS) and Surface-Enhanced Laser Desorption/Ionization (SELDI)-MS coupled to fractionation techniques like Capillary Electrophoresis (CE) or Liquid Chromatography (LC). 2DE is one of the most widely used separation methods having some advantages such as being robust, which can be implemented in most laboratories [14]. Using this method, proteins are separated in two dimensions based on their isoelectric point and molecular weight. In 2D Differential Gel Electrophoresis (2D-DIGE), various dyes are implemented for each sample, and then the samples are mixed and separated on the gel. This method is labor and time saving compared to 2DE and produces more reliable results [15]. Mass spectrometry provides rich information about proteins,and is capable of detecting thousands of peptides in a single separation [16]. Mass-based approaches are well suited in terms of sensitivity and throughput [17]. MALDI mass imaging is another mass-based technique established as a robust tool for spatially resolved analysis of biomolecules directly *in -situ* with high resolution and high throughput [18]. As - accurate quantitation of proteins is a key issue in proteomic biomarker discovery, quantitative approaches such as stable isotope labeling methods have emerged. In these methods, protein-containing samples are labeled with different stable isotopes; they are mixed, and then are subjected to LC-MS analysis. The most widely used isotope labeling techniques include Isotope-Coded Affinity Tag (ICAT), Stable Isotope Labeling by/with Amino Acids in cell culture (SILAC), and isobaric Tags for Relative and Absolute Quantitation (iTRAQ). iTRAQ is a suitable method for biomarker discovery, since it provides high sequence recovery and direct identification of biomarkers through analysis of mass spectra, owing to isobaric tags, although there are some limitations regarding its application including limited resolution or low throughput [19]. Tandem Mass Tag (TMT) coupled to mass spectrometry is also a labeling quantitative approach, enabling accurate comparison of multiple samples at a same time [20]. Development of metabolomic biomarker discovery also relies on improvement of resolution power of analytical techniques such as Liquid and Gas Chromatography (LC and GC) in combination with mass spectrometry methods and Nuclear Magnetic Resonance (NMR) spectrometry. Due to preferences and limitations of these techniques, they should be used as complementary methods, which would provide a wider range of metabolites to be identified. NMR has high reproducibility and requires minimum sample pretreatments, where mass-based methods are highly sensitive and selective, but they require sample destruction and more pretreatment steps [21]. Due to importance of proteomic and metabolomic approaches in biomarker discovery of glomerular disorders, this study was conducted to review an outstanding number of metabolites and proteins that have recently been identified and suggested as potential biomarkers of a number of CKD_s_.

### Overview of a number of high-risk CKDs

Membranous Glomerulonephritis (MGN), *Focal Segmental Glomerulosclerosis* (FSGS), and immunoglobulin-A Nephropathy (IgAN) are three types of CKDs, and a considerable percentage of patients with these diseases eventually reach ESRD [22–24].

#### Membranous glomerulonephritis

MGN is the most common primary cause of nephrotic syndrome characterized by immune deposits in the subepithelial space and the Glomerular Basement Membrane (GBM) thickening [25]. Up to 40% of MGN patients reach ESRD [22]. Deposited antibody belongs to Immunoglobulin G (IgG) class. In 30% of cases, IgM and IgA, produced following secondary MGN have also been observed. In 75% of cases, complement component 3 (C3) and C5b-C9 have been reported in urinary sediments [25]. MGN accounts for about 25% of kidney biopsies done on patients with renal diseases [26, 27]. There are two types of MGN: primary and secondary. Secondary form appears after a systematic disorder [27] such as infectious diseases (e.g., hepatitis B and C), drugs and toxins (e.g., penicillamine), autoimmune or collagen-vascular diseases (e.g., Systemic Lupus Erythematosus [SLE]), neoplastic diseases (e.g., carcinomas), post-renal transplant glomerulopathy, and miscellaneous conditions (e.g., diabetes mellitus). In 75–80% of cases, no correlation has been reported with any systematic disease. Primary form is also called idiopathic MGN [26, 27] mostly observed in adult males. People aged between 30 and 50 years old are more likely to be affected with primary MGN. In recent years, some potent molecules have been reported as new candidates for inducing primary MGN, such as aldose reductase, superoxide dismutase, α-enolase, neutral endopeptidase, and thrombospondin type-1 domain-containing 7A protein [28]. In secondary MGN, different antigens have been identified as effective factors contributing to the disease such as antigen e (in hepatitis B), double -stranded DNA (in SLE), carcinoembryonic antigen (in colon cancer),and thyroglobulin antigen (in Hashimoto’s thyroiditis) [25].

**Fig. 1:**
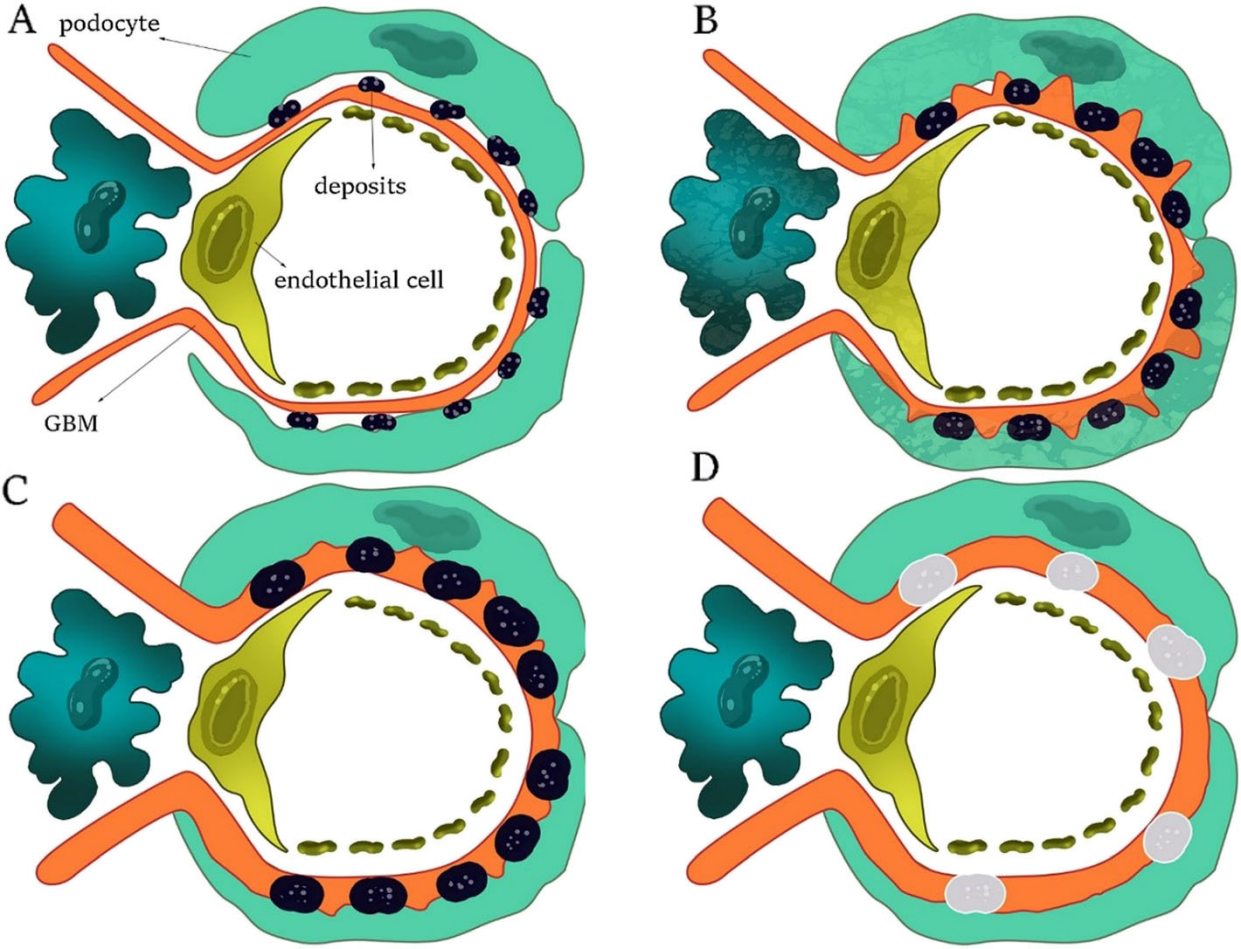
The progression of MGN: (**a**) Stage Ι. (**b**) Stage ΙΙ. (**c**) Stage ΙΙΙ. (**d**) Stage ΙV. The images are a schematic from inside the glomeruli

Effective antigen inducing primary MGN in humans was unknown for a long time. After many laborious experiments, Phospholipase-A2-Receptor (*PLA2R*) was finally identified as an antigen, potentially playing an important role in occurrence of primary MGN. In summary, the PLA2R was discovered by western blotting method under non-reducing condition. Using this protocol, disulfide bonds and *epitope structures of the PLA2R are preserved, and* antigen detection is possible due to its antibody [27]. Recent studies have reported different epitope structures of PLA2R in patients with primary MGN. This has been done using a combination of structural genetic engineering, mass spectrometry, and crystallography [27, 29, 30]. With the advancement of science, engineering of small molecules in order to bind cysteine-rich part of the PLA2R (and stopping formation of disulfide bonds) aimed at treating primary MGN is not impossible [27]. However, it must be noted that, PLA2R justifies only 80% of primary MGNs. In recent years, other potent molecules have been reported as new candidates for inducing primary MGN, such as aldose reductase, superoxide dismutase, α-enolase, and neutral endopeptidase [27, 31]. In MGN, complement complex C5b-C9, also known as Membrane Attack Complex (MAC), damages glomerular epithelial cell membranes, enters through the cells, and induces sublytic corruption. In 80% of cases, excessive proteinuria is observed at early stages of the disease. Hypertension mostly occurs after renal insufficiency, but rarely occurring at the time of presentation (30% of cases). Microscopic hematuria is observed in 50% of cases, and urinary C5b-C9 increases in some patients. Due to occurrence of hypercoagulable state in MGN, risk of fatal pulmonary embolism remains, which is similar to other types of nephrotic syndrome [25]. MGN includes four stages (Fig. 1):

##### Stage Ι

Few scattered deposits can be seen in the subepithelial space. The GBM has not been thickened yet. It is discernible by immunofluorescence (staining either IgG or PLA2R) and electron microscopy (electron-dense deposits along the glomerular capillary wall) [25, 27].

##### Stage ΙΙ

Deposits have increased and become larger. The GBM raises itself from the spaces among the deposits and creates sharp structures called “spikes.” The GBM begins to thicken and can be identified by silver staining.

##### Stage ΙΙΙ

Deposits have become larger compared to previous stage. The GBM has covered the sediments and has fully thickened. Spikes are still visible by silver staining. Granular deposits can be seen by immunofluorescence at stages ΙΙ and ΙΙΙ.

##### Stage ΙV

The sediments gradually disappear, but the GBM is still thick [25].

#### Focal segmental Glomerulosclerosis

**Fig. 2:**
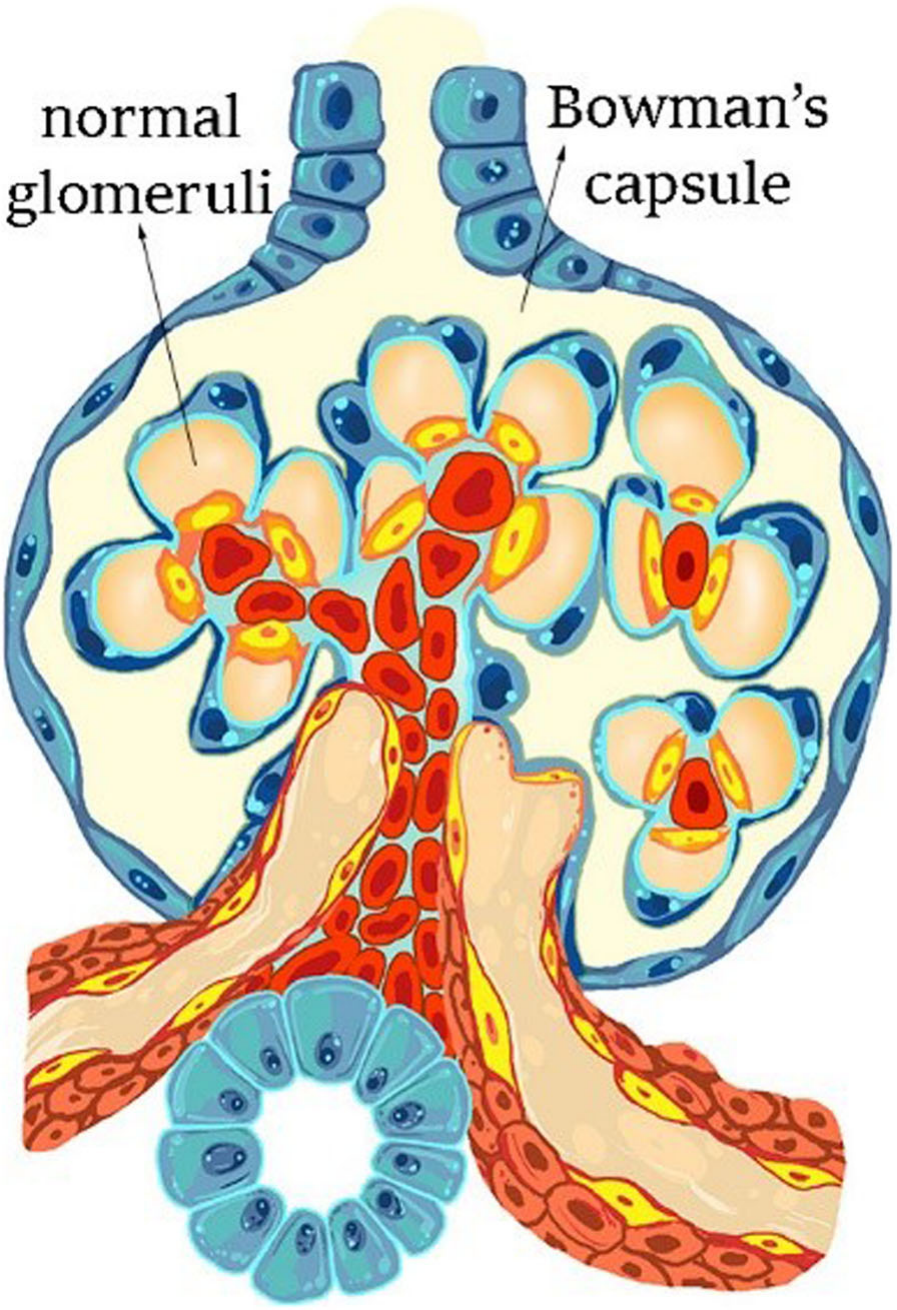
The normal glomerulus in Bowman’s capsule is shown with no signs of sclerosis

**Fig. 3:**
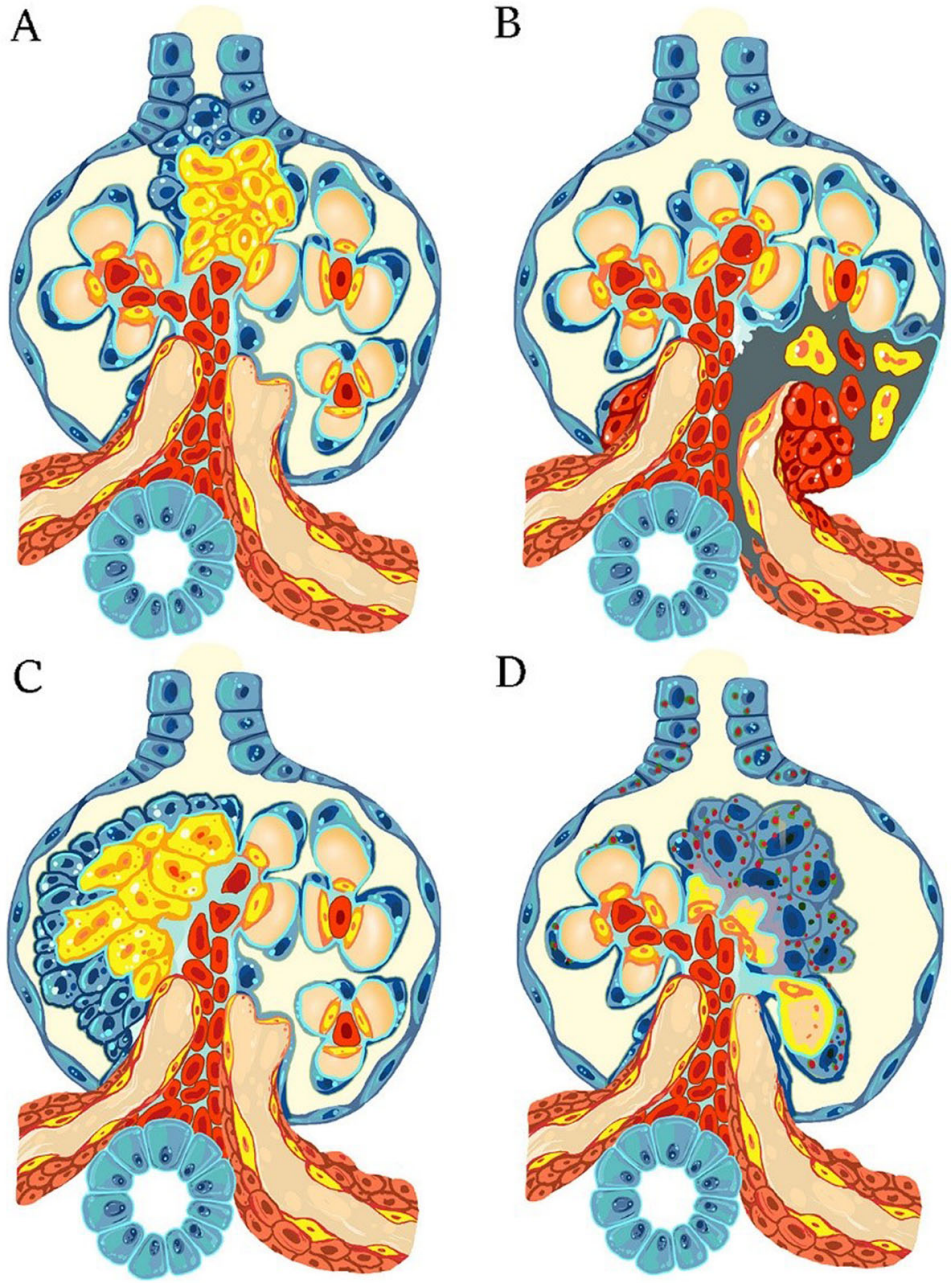
Four different subtypes of FSGS are shown according to the columbia classification. (**a**) Tip lesion FSGS. (**b**) Perihilar FSGS. (**c**) Cellular FSGS. (**d**) Collapsing FSGS

FSGS is mostly accompanied by nephrotic-range proteinuria. Destruction of glomerular capillary loops and an increase in extracellular matrix occur in some of the glomeruli, but sclerosis does not include all parts of the glomeruli. According to the Columbia Classification, FSGS is divided into five subtypes of cellular, perihilar, collapsing, tip lesion, and not otherwise specified morphologic forms with respect to the pattern and intraglomerular distribution of sclerotic lesions (Fig. 2 and Fig. 3). The evidence suggests that, podocyte damage is definite at the time of presentation and progression of the disease [32]. Podocytes are one of main agents involved in maintenance of a normal filtration barrier [33]. TGF-β causes nephrin to be released into the podocytes. Nephrin acts as a transcription factor in the cell inducing more expression of cathepsin L; subsequently, CD2-Associated Protein (CD2AP) is defragmented [33]. There are two types of FSGS: primary and secondary [32, 33]. In primary form, podocytes are directly injured [32] and reduction of renal function occurs quickly [33]. In secondary form, however, many factors could help induce the disease (for example, kidney mass reduction, obesity, pharmaceutical toxicity, viral infection, genetic background, hypertension-related diseases, and pyelonephritis) [32]. In addition, secondary form has a slow progression procedure. Thus, 35% of patients with nephrotic syndrome having sustained renal biopsy fall into the category of FSGS. Prevalence of the disease in males is 1.5- - 2-fold higher than females [33].

It seems that some molecules are involved in induction of primary FSGS and its recurrence after transplantation; known as circulating permeability factors [32]. Three kinds of Circulating Factors (CF) have been found so far including soluble urokinase-type Plasminogen Activator Receptor (suPAR), Cardiotrophin-Like Cytokine Factor 1 (CLCF1), and anti-CD40 [32, 33]. Savinet, et al. extracted CLCF1 from the serum of patients with FSGS. They injected CLCF1 into rat model, which led to an increased albuminuria [32, 34]. They estimated molecular weight as 22 kDa [33], and it had a high affinity for connecting with galactose, Janus Kinase 2 (JAK2), and Signal Transducer and Activator of Tanscription 3 (STAT3) inhibitors. Given that combination of CLCF1-galactose is easily removed by the liver, the effect of galactose treatment on patients with FSGS needs to be taken into account [32, 34]. Future studies are suggested to focus on application of JAK2 and STAT3 inhibitors in treatment of FSGS [33, 35].

It has been reported that, serum level of suPAR is high in primary FSGS as well as in recurrence of the disease after renal transplantation [32, 36]. A significant correlation has also been found between having a serum level of suPAR over 3000 pg/ml and incidence of primary FSGS and post-transplant recurrence of FSGS in humans [33, 37]. It has also been reported that, suPAR has a negative correlation with estimated GFR and treatment response in FSGS [32, 37]. Huang, et al. showed that, level of suPAR increases in the serum of patients with FSGS compared to MGN and MCD [38]. In mouse models, suPAR increased αvβ3 integrin activity; accordingly resulting in more stimulation of foot processing in the podocytes, as well as more proteinuria [33, 39]. Concerning anti-CD40, studies have shown that, the interaction between suPAR and αvβ3 integrin induces Post-Translation Modification (PTM) in CD-40 protein in the podocytes; then, a new epitope appears on extracellular domain of the molecule as a result of which anti-CD40 is secreted. In -vitro studies have indicated that, anti-CD40 is able to damage actin protein in the podocytes [33, 40].

Considering the role of CFs in pathogenesis of FSGS, using CF inhibitors may provide new therapeutic options in the future [33, 41]. There is a theory suggesting that, FSGS could occur due to mutation on the genes interfering with the structures of the slit diaphragm, actin in the podocytes cytoskeleton, and foot processing of podocytes on the GBM [32, 42–44]. For example, a mutation in the NPHS1 gene leads to disruption in expression of nephrin; subsequently leads to occurrence of hereditary nephritic syndrome [32]. The WT1 gene regulates transcription of NPHS1 [32, 45].

*Activated Parietal Epithelial Cells* (PECs) with CD133 and CD24 markers can migrate from their original site and replace injured podocytes by creating an adhesive site between the glomerular tuft and Bowman’s capsule, but they can also induce sclerosis in the glomeruli [32, 46–49]. Recent studies have suggested CD44 as a marker of activated PECs, and these cells can be distinguished at early primary and early post-transplant recurrence of FSGS [32, 49].

#### IgA nephropathy

**Fig. 4:**
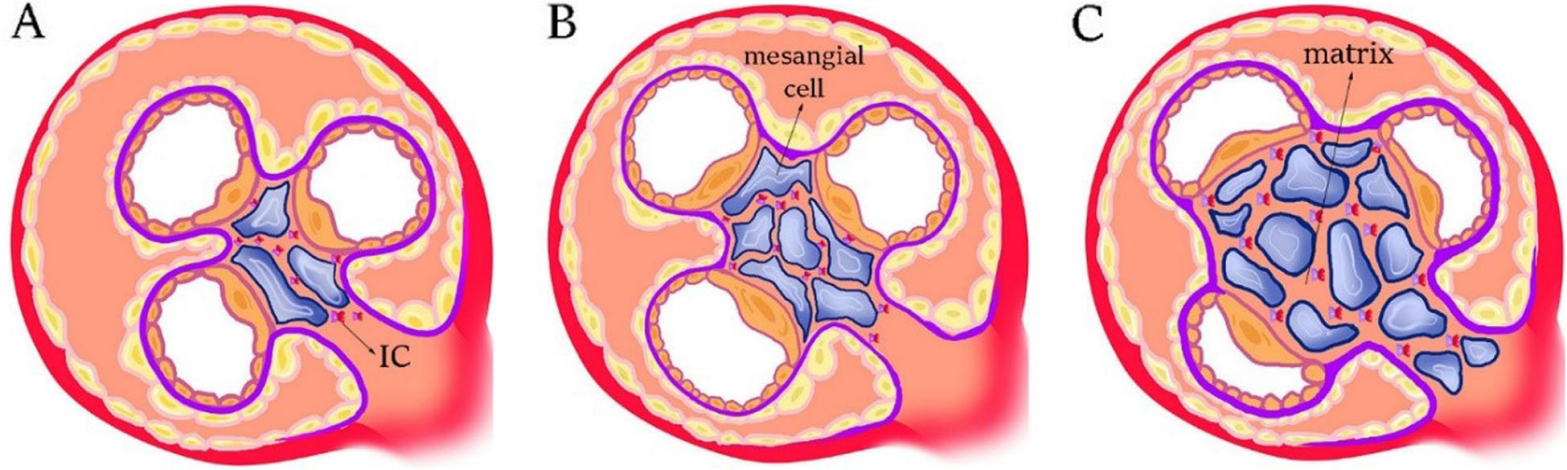
The schematic images representing the progression of disease in IgAN. (**a**) The initial stage of the disease by deposition of immune complexes in the mesangial area of Bowman’s capsule. (**b**) Proliferation of the mesangial cells. (**c**) Proliferation of mesangial cells accompanied by matrix expansion

IgAN is recognized by mesangial deposition of Immune Complexes (ICs) [50]. Histopathologically, the disease is identified by expansion of mesangial matrix and proliferation of mesangial cells, and/or mononuclear cell infiltration in mesangial region (Fig. 4). In mesangial region, C3 is observable along with ICs. Periodic acid-shift staining shows proliferation of mesangial cells and expansion of mesangial matrix. In addition, electron density increases in mesangial areas [2]. Up to 50% of patients with IgAN experience ESRD as the disease progresses [50]. In Japan, 40–60% of patients with chronic glomerulonephritis suffer from IgAN, which is the most common cause of primary chronic glomerulonephritis in the world [2]. IgA-containing ICs are formed in circulation of blood, and some of them are deposited in renal tissue causing glomerular damage [50, 51]. Pathogenic IgA in ICs belongs to the IgA1 subclass, in which some of glycan fragments linked (O-linked glycosylation) to the Hinge Region (HR) of antibody do not contain galactose (Gal); therefore, these antibodies are called Galactose-deficient IgA1 (Gd-IgA1) [50]. Gd-IgA1 antibodies are almost polymeric (pGd-IgA1) in mesangial area [2]. A series of bacterial enzymes, called IgA1-specific proteases are able to specifically cleave the HR in IgA1 [50, 52] found in some respiratory pathogens. These enzymes, therefore, have been considered for therapeutic goals. Macroscopic hematuria is a common symptom of the disease, and sometimes the disease is associated with upper-respiratory and/or gastrointestinal tract infections [50]. Some scientists believe that, mucosal membrane could be origin of Gd-IgA1 production and secretion after inflammation of mucosal membrane due to the pathogens [50, 53]. Accordingly, expression and/or activity of glycosyltransferases are changed in the IgA1-secreting cells. After forming Gd-IgA1, the anti-IgA1 antibody is excreted against Gd-IgA1. These antibodies mostly belong to the IgG class. The connection between anti-IgA1 and Gd-IgA1 and formation of immune complexes occurs in circulation of patients with IgAN. These ICs are able to activate mesangial cells, inducing cellular proliferation, and leading to more secretion of cytokines/chemokines and extracellular matrix compounds, in -vitro. However, uncomplexed Gd-IgA1 cannot induce proliferation of mesangial cells by itself. A positive correlation has also been found between circulatory level of pathogenic ICs and disease activity; therefore, serum level of Gd-IgA1 and anti-IgA1 can be used to predict disease progression and its post-transplant recurrence [50]. It is possible to measure serum level of Gd-IgA1 using ELISA (code no. 27600) commercially [2]. Recently, four clinical markers have been reported for diagnosis of IgAN (or for differential diagnosis of the disease from the other types of primary chronic glomerulonephritis); which are as follows: [1] > 5 RBCs/HPF in urinary sediments, [2] persistent proteinuria (urinary protein > 0.3 g/d), [3] a serum IgA level of > 315 mg/dl, and [4] a serum IgA/C3 ratio of > 3.01 [2]. Chan, et al. found that, IC deposition in mesangial region leads to secretion of Tumor Necrosis Factor-α (TNF-α) by mesangial cells; they also found that, inflammatory changes occur in renal interstitium [2, 54]. Subsequent research showed a high level of TNF pathway-related molecules, such as TNF-α and TNF receptors (TNFRs) in the serum of patients with IgAN [2]. Podocalyxin is a glycoprotein located in membrane of podocytes. In adults with IgAN, glomerulosclerosis seems to be induced due to podocyte damage. Asao, et al. demonstrated that, level of podocalyxin and number of podocyte cells in the urine of adult patients with IgAN is correlated with severity of glomerular injury. Therefore, these measures can be used as biomarkers to predict histological changes in the adults with IgAN [2, 55]. As mentioned earlier, in many cases, IgAN follows upper respiratory tract infections; therefore, the correlation between tonsillar infection and IgAN has been of interest to the researchers. A tonsil is a mucosal lymphatic organ, and polymeric IgA1 is produced on its mucosal membrane by plasma cells located in the tonsil. Some pIgA1 molecules seem to be involved in pathogenesis of IgAN. Nakata, et al. showed that, palatine tonsils are main producer of Gd-IgA1. In a new therapeutic approach devised for IgA patients in Japan, a tonsillectomy is used along with steroid pulse therapy. However, in some patients, plasma cells migrate from the tonsils into another organ; therefore, a tonsillectomy alone is not a proper therapeutic response [2, 56–59]. Three glycosyltransferases have been found to be increased in Gd-IgA1-secreting cells: core 1 β1,3-galactosyltransferase (C1GalT1), α-N-acetylgalactosaminide α-2,6 sialyltransferase 2 (ST6GalNAc-ΙΙ), and N-acetylgalactosaminyltransferase 14 (GalNAcT14) [50, 60–64]. Recently, it has been discovered that, IL-6 and IL-4 induce expression level of C1GalT1 and ST6GalNAc-ΙΙ, leading to more formation of Gd-IgA1 [50, 53]. Therefore, restricting production of IL-6 and IL-4 through inhibition of Jak-STAT pathway could be beneficial in treatment of IgAN [50, 65].

It should be noted that, anti-IgA1 binds to HR of the Gd-IgA1 molecule [50]. Some scientists believe that, recombinant synthetic HR fragments or any other peptides enzymatically bound to N-Acetylgalactosamine (GalNAc) can be produced to prevent formation of ICs in patients with IgAN [50, 51, 66]. ICs activate mesangial cells by phosphorylation of a wide range of proteins. Therefore, using some specific protein kinase inhibitors might prevent progression of renal damage in patients with IgA.N [50, 67].

### Proteomic studies in case of FSGS, MGN, and IgAN

Modern technologies such as “proteomics” have led to new opportunities in searching for biomarkers of renal diseases especially in bio-fluids, mostly in urine, serum, and plasma, which are much less invasive for the patients. Urine sample prepared by a non-invasive collecting method offers a valuable source of biomarkers, reflecting physiological state of the system. Except for the urine itself, urinary microvesicles or exosomes are also rich in biomarkers. However, some considerations must be taken into account such as time of sampling, as urine chemical content varies during different times of a day.

#### Membranous glomerulonephritis

In a recent study, Smith, et al. investigated two groups of patients with MGN, who were responsive and non-responsive to immunosuppressive therapy, using MALDI-MS imaging, in order to find therapeutic markers of the disease. They proposed two proteins including Sonic Hedgehog (SHH) and α-Smooth Muscle Actin (α-SMA) as putative tissue biomarkers to differentiate Responders (R) and Non-Responders (NR) [18]. Higher SHH signal in the NR group may reflect activation of SHH pathway leading to development of glomerular lesion. In addition, Α-SMA has been reported to be correlated with different kinds of glomerulonephritis [68, 69]. Pang, et al. introduced some urinary proteomic markers for primary MGN using a Tandem Mass Tag (TMT) and a nano-scale Liquid Chromatography coupled to tandem Mass Spectrometry (LC-MS/MS). Among 249 identified proteins, α-1-antitrypsin (A1AT) and afamin (AFM) have been confirmed by western blotting analysis. Overexpression of A1AT has been shown to inhibit neutrophil elastase contributing to glomerular integrity [70]. Role of AFM in progression of glomerular diseases is not clear, however, it has been introduced as a putative marker of FSGS and IgAN in previous studies [71, 72]. Gene ontology analysis showed that, platelet degranulation, calcium ion binding, and extracellular exosome, respectively are the most significant biological process, molecular function, and cellular component related to MGN. “Complement and coagulation cascades” have also been suggested as the most important KEGG pathways in MGN pathology [73]. In another study, Bruschi, et al. identified the antigens of autoantibodies in patients with MGN by an “in -vivo” proteomic method. They resolved podocyte proteins by 2DE followed by semidry blotting to nitrocellulose membrane. They used antibodies in serum and micro-dissected glomeruli to detect podocyte antigens characterized by mass spectrometry. Using this approach, they obtained 6 new proteins including α-enolase, superoxide dismutase 2, vimentin, aldolase reductase, glycine-tRNA ligase, and elongation factor 2 in MGN [74]. Sui, et al. also investigated potential biomarkers of MGN and characterized those proteins according to Gene Ontology (GO). They compared tissue protein profiles of patients to that of healthy subjects by means of iTRAQ method followed by MALDI-TOF-TOF mass spectrometry. Then, they mainly mapped 423 differentially expressed proteins related to immune response, immune effector process, positive regulation of immune system process, and activation of immune response [75]. Given that, MGN is an autoimmune disease accompanied with accumulation of immune deposits on basement membrane of glomeruli, it seems logical that, humoral and cellular immune mechanisms play a fundamental role in pathogenesis of the disease. Among identified proteins, β2-microglobulin has been shown to be a specific and accurate prognostic factor for MGN directly associated with regulation of immune processes. Ligabue, et al. proposed that, interstitial fluid could be considered as a valuable source of biomarkers in patients with idiopathic MGN. They obtained interstitial fluid from renal biopsy samples and performed a proteomic study using an ESI-Q-TOF mass spectrometer followed by confirmation of the results by immunofluorescence analysis of renal tissues and western blotting analysis of urine, serum, and podocyte cell lines. They observed, for the first time, an increase in expression of PDLI5 and LDB3 in kidney podocytes, both belonging to the LIM domain-related proteins [76]. Expression of these proteins in podocytes cytoskeleton might be related to plasticity of actin cytoskeleton or some podocyte dysfunctions as a result of proteinuria [77]. Rood, et al. studied urinary microvesicles proteome as an important source of intracellular and membrane-bound proteins in patients with idiopathic MGN (iMGN) compared to those with idiopathic FSGS (iFSGS) and healthy controls. They applied iTRAQ labeling followed by LC-MS/MS analysis. They confirmed significant up-regulation of Lysosomal Integral Membrane Protein type 2 (LIMP-2) in renal biopsy of the patients with iMGN compared to those with iFSGS, IgAN, MCD, membranoproliferative glomerulonephritis (MPG) and healthy controls using immunofluorescence [78]. Overexpression of LIMP2 might be due to protein transport dysfunction between lysosomes and the podocytes plasma membrane, as LIMP2 plays a key role in biogenesis and maintenance of lysosomes and endosomes. Therefore, intracellular proteins are expressed on podocytes membranes [79, 80]. Smith et al. used MALDI-MS imaging for FFPE renal biopsy samples prepared from 20 patients with primary and secondary MGN. They detected serine/threonine protein kinase MRCK gamma with a signal of 1459 m/z as the most significant distinguishing protein between primary and secondary MGN. They could also detect PLA2R, IgG4, and m/z signals of 1094, 1116, 1459, and 138, differentiating these two groups. They also studied the samples obtained from patients with FSGS, IgAN, and MCD and compared them with those of healthy controls most of which were positive to PLA2R and IgG4 [81]. The role of MRCK gamma in pathogenesis of MGN is not fully understood; however, it might alter actin dynamics and influence podocytes foot processes following phosphorylation of downstream proteins. Consequently, these processes will lead to glomerular dysfunction and development of nephrotic syndrome [82]. Ngai, et al. performed a gel-based approach to find candidate biomarkers of MGN in Sprague-Dawley rat models. They collected urine samples at serial intervals of 0, 10, 20, 30, 40, and 50 days following induction of the disease. They studied protein profiles by 2D-PAGE and identified them by MALDI-TOF-MS. They found some proteins, which were considered as potential diagnostic biomarkers of MGN. These proteins included progesterone-induced blocking factor 1 isoform a, ribosomal protein L5, vimentin, tropomyosin isoform 6, α-1-antitrypsin, serotransferrin precursor, Ba1–647, haptoglobin precursor, preprohaptoglobin, serum albumin precursor, CTP: phosphocholine cytidylyl transferase, anti-idiotype immunoglobulin M light chain, and vascular endothelial growth factor -A,a splice variant of VEGF 102 [83]. Among identified proteins, some of them have been previously reported to have a role in pathogenesis of glomerulopathies. α-1-antitrypsin is a serine protease inhibitor suggested as a marker of glomerular diseases in previous studies with regulatory function in inflammatory processes [84, 85]. Altered expression of VEGF might be related to its role in enhancing TGF-β expression in glomerular endothelial cells via MAPK and PI3K pathways, influencing permeability of blood vessels [86]. Details on the most important identified proteins are presented in Table 1.

**Table 1:** Proteomic studies in the case of FSGS, MGN and IgAN

CKD type	Sample Type	Method	Putative Biomarkers	Ref.
MGN	Tissue	MALDI-MS imaging	sonic Hedgehog (SHH), α-smooth muscle actin (α-SMA)	(Smith et al., 2019)
	Urine	tandem mass tag (TMT), nano-scale LC-MS/MS	Alpha-1-antitrypsin, Serotransferrin, Ceruloplasmin, Serum albumin, Carbonic anhydrase 1, Transthyretin, Haptoglobin, Leucine-rich alpha-2-glycoprotein, Alpha-1B-glycoprotein, Afamin	(Pang et al., 2018)
	Serum, Tissue	2DE, semidry blotting, Mass	α-enolase, superoxide dismutase 2, vimentin, aldolase reductase, glycine-tRNA ligase, elongation factor 2	(Bruschi et al., 2015)
	Urine	2DE, MALDI-TOF-MS	progesterone-induced blocking factor 1 isoform a, ribosomal protein L5, vimentin, tropomyosin isoform 6, α-1-antitrypsin, serotransferrin precursor, Ba1–647, haptoglobin precursor, preprohaptoglobin, serum albumin precursor, CTP:phosphocholine cytidylyl transferase, anti-idiotype immunoglobulin M light chain, vascular endothelial growth factor A splice variant VEGF 102	(Ngai et al., 2006)
	Serum	SDS-PAGE	29 KD, 5 KD	(Pant et al., 2016)
	Tissue	iTRAQ, MALDI-TOF-TOF-MS	C1RL, TUBB, CD74, CORO1A, B2M, KRT1, ARHGDIB, HLA-DRB1, HLA-A, HLA-DRB1, IGHG1, KV102, ERAP1, MYLPF, GBP1, HLA-B, FTH1, VCAM1, C4BPA, HSPD1, C1QBP	(Sui et al., 2015)
	Interstitial Fluid, Tissue, Urine, Serum, Podocyte Cell Lines	ESI-Q-TOF-MS, immunofluorescence	PDLI5, LDB3	(Ligabue et al., 2013)
	Urinary Microvesicles, Tissue	iTRAQ labeling and LC-MS/MS, immunofluorescence	LIMP2, TPP1, AMBP, PDZK1P1, DCHS2, MGAM, SLC9A3R1, ATAD5, HYDIN, MYO15A, C7, MUC1, SDCBP, GPRC5B, ATG2B, RAC1, EHMT1, RHOC, KMT2B, EPS8, EVC2, SLC44A2, SLC12A3, MYO9B, CCDC81	(Rood et al., 2015)
	Tissue	MALDI-MS imaging	serine/threonine protein kinase MRCK gamma, PLA2R, IgG4	(Smith et al., 2017)
IgAN	Tissue	immunostaining	CAP1, PRCP, SHC1	(Krochmal et al., 2017)
	Urine	IEF/LC-MS/MS	AFM, ALB, APOA1, AZGP1, A2M, CA1, CP, C3, C4A, FN1, F2, GC, HP, IGFBP7, PSAP, SERPINA1, SERPINA3	(Mucha et al., 2014)
	Urine	nanoscale LC-high resolution tandem MS	transferrin, α1-antitrypsin, albumin fragments, fibulin-5, prasoposin, YIP1 family member 3, osteopontin	(Mohammadi Majd et al., 2018)
	Urine	2D-LC-MS/MS, iTRAQ, Western blot	ICAM1, TIMP1, SERPINC1, ADIPOQ	(Guo et al., 2018)
	Urine	LC-MALDI-TOF/MS, proteins arrays, immunomagnetic isolation	IgA-uromodulin complex, α1-antitrypsin, galactose-deficient IgA1	(Neprasova et al., 2016)
	Tissue	MALDI-MS imaging	m/z of 4963, 5072	(Smith et al., 2016)
	Urine	nano-LC-MS/MS	CD44, glycoprotein 2, vasorin, protocadherin, epidermal growth factor, CLM9, dipeptidyl peptidase IV, NHL repeat-containing protein 3, utreoglobin, SLAM family member 5	(Samavat et al., 2015)
	Urine	SELDI-TOF-MS, MALDI-TOF-MS	perlecan laminin G-like peptide, IgK light chains	(Rocchetti et al., 2013)
	Urine, Serum, Plasma	magnetic bead technology, MALDI-TOF-MS	uromodulin, bradykinin and α-1-antitrypsin urine m/z:1769, 1898, 1913, 1945, 2491, 2756, 2977, 3004, 3389, 4752 blood m/z:2953, 5337, 9287, 9289	(Graterol et al., 2013)
	Urine	MALDI-TOF-MS	m/z: 2740, 3038, 2747, 3032, 3235, 2095, 3328, 6174, 6189, 2754	(He et al., 2012)
	Urine	2DE, nano-HPLC-ESI-MS/MS	kininogen, inter-α-trypsin-inhibitor heavy chain 4, tranthyretin	(Rocchetti et al., 2008)
	Urine	2DE, MALDI-TOF-MS	A panel of 59 protein markers	(Park et al., 2006)
	Urine	2D-DIGE, LC-MS/MS	α1-microglobulin, transferrin, albumin, retinol-binding protein 4, β-globin, carbonic anhydrase I, cystatin C, α2-glycoprotein 1	(Yokota et al., 2007)
	Urinary Exosomes	LC-MS/MS	aminopeptidase N, α-1-antitrypsin, vasorin precursor, ceruloplasmin	(Moon et al., 2011)
	Urine	2DE, MALDI-TOF-TOF/MS, western blot	α-1-antitrypsin, albumin fragments, α-1-β-glycoprotein, laminin G-like fragment of endorepellin	(Surin et al., 2013)
	Urine	CE-ESI-TOF-MS	A panel of polypeptides molecular weights	(Haubitz et al., 2005)
	Urine	nanoflow LC-MS/MS, GeLC-MS/MS	afamin, leucine-rich alpha-2-glycoprotein, ceruloplasmin, alpha-1-microgolbulin, hemopexin, apolipoprotein A-I, complement C3, vitamin D-binding protein, beta-2-microglobulin, retinol-binding protein 4	(Kalantari et al. 2013)
FSGS	Urine	nano-flow LC-MS/MS	RNAS2, CD59, PTGDS, B2MG, AMBP, SULF2, CBG, AFAM, MXRA8, CO6A1, ACTG, HPT	(S. Kalantari et al., 2014)
	Urine	LC-MS/MS, Western blot	serum albumin, serotransferrin, α-1-1antiproteinase, afamin, ceruloplasmin, plasminogen, AMBP	(Zhao et al., 2014)
	Urine	magnetic bead-based technology, MALDI-TOF/MS	Uromodulin, α-1-antitrypsin fragment	(Perez et al., 2014)
	Urine	nano-LC-MS/MS	apolipoprotein-A1, matrix-remodeling protein 8	(Shiva Kalantari et al., 2014)
	Urine	2D-DIGE, MALDI-TOF MS	α-1-antitrypsin, transferrin, 39S ribosomal protein L17, histatin-3, calretinin	(Perez et al., 2017)
	Urine	2DE, MALDI-TOF/MS	glutathione S-transferase, collagen IV fragment, E-cadherin	(Shui et al., 2008)
	Urine	2DE, tandem-MS	UBA52, CD74, SKP1, CXADR, S100A13, SYNE1, PVALB, FRY, GSTA3, CROCC, LRBA, DAB2	(Wang et al., 2017)
	Serum	SDS-PAGE	29 KD, 5 KD	(Pant et al., 2016)
	Tissue	MALDI-MS imaging	m/z of 4025, 4048	(Smith et al., 2016)
	Urine	nano-flow LC-MS/MS	CD59, CD44, IBP7, DPEP1, Robo4	(Nafar et al., 2014)

*MGN* Membranous glomerulonephritis, *IgAN* Immunoglobulin A nephropathy, *FSGS* Focal segmental glomerulosclerosis, *MCD* Minimal change disease, *iFSGS* idiopathic Focal segmental glomerulosclerosis, *iMGN* idiopathic Membranous glomerulonephritis, *MPG* Membranoproliferative glomerulonephritis, *DN* Diabetic nephropathy, *LN* Lupus nephritis, *TBMN* Thin-basement membranous nephropathy

#### Focal segmental Glomerulosclerosis

Kalantari, et al. evaluated prognostic protein markers between two groups of FSGS patients with mild (with eGFR> 60 cc/min/1.73m^2^) and advanced (with eGFR< 60 cc/min/1.73m^2^) states of disease. They identified urine proteome using nano-flow LC-MS/MS method. Among 54 significantly altered proteins, ribonuclease 2 and haptoglobin were the most important in terms of fold change. Complement and coagulation cascade was also the most significant pathway shown to be impaired in advanced stage of the disease [87]. Pathologic role of ribonuclease 2 in glomerulosclerosis is not clear, however, a correlation has been reported between its serum level and renal insufficiency [88]. A correlation has also been reported between haptoglobin and renal function [89]. Zhao, et al. investigated urinary proteome in Adriamycin-induced rat models of FSGS. They profiled urine samples using LC-MS/MS and identified 23 altered proteins, 20 of which had human orthologs where 13 of them could be detected in normal human urine. Among 20 proteins, 7 of them were selected and verified by western blotting analysis including serum albumin, serotransferrin, α-1-antiproteinase, afamin, ceruloplasmin, plasminogen, and AMBP [71]. Among 20 proteins, albumin, serotransferrin, and kininogen-1 have been previously reported to be increased in FSGS [90, 91]. In another study, Pérez, et al. used a 2D-DIGE method coupled with MALDI-TOF-MS proteomic approach to discover urinary biomarkers differentiating FSGS and Minimal Change Disease (MCD), as FSGS is usually resistant to steroid therapy whereas MCD is responsive. They divided 49 patients randomly into a training (11 FSGS, 10 MCD) set and a validation (14 FSGS, 14 MCD) set. They quantified identified proteins from the training set in the urine samples of the validation set by ELISA method. The most significant alterations included decrement of α-1 antitrypsin, transferrin, 39S ribosomal protein L17, and histatin-3, and increased calretinin in FSGS patients compared to MCD [6]. Among these proteins, the role of α-1 antitrypsin in FSGS pathogenesis might be related to its anti-inflammatory and anti-apoptotic actions, as previously shown in renal injuries [92]. Increased urinary excretion of transferrin is also correlated with severity of glomerulosclerosis [93]. Kalantari, et al. also investigated predictive biomarkers related to responsiveness of FSGS patients to steroid drugs using a urinary proteomics approach. They compared 6 steroid-sensitive and 4 steroid-resistant patients and identified protein profiles by means of a nano-LC-MS/MS system followed by multivariate statistical analysis. Among 22 differentially expressed proteins, apolipoprotein-A1 (APOA1) and matrix-remodeling protein 8 (MXRA8) were the most significant [94]. Overexpression of APOA1 may be related to its role in inhibition of LDL oxidation during atherosclerotic process. Significant decrease in MXRA8 level in non-responders might be due to higher prevalence of interstitial fibrosis in this group of patients [95].

Pant, et al. studied diagnostic markers of FSGS and primary MGN using SDS-PAGE analysis of serum samples separating proteins according to molecular weight. They categorized patients according to their proteinuria levels into 3 groups: mild (proteinuria< 4 g/24 h), moderate (4 g/24 h < proteinuria< 8 g/24 h), and severe (proteinuria> 8 g/24 h). They showed that, content of the 29 KD protein was significantly higher in FSGS compared to MGN, where the 5 KD protein was much higher in severe and moderate proteinuria than mild [96]. They proposed that, the 29 KD protein might correspond to suPAR or apolipoprotein A. They also proposed that, the 5 KD protein may contribute to severity of the disease, although these proteins must be characterized by other methods. Shui, et al. searched for potential diagnostic and prognostic FSGS pre-sclerotic and serial sclerotic stages biomarkers in urine samples obtained from FSGS mouse models. They used 2DE method combined with MALDI-TOF/MS and identified 37 proteins with dynamic changes during disease progression. Among these proteins, glutathione S-transferase, collagen IV fragment, and E-cadherin were confirmed by western blotting analysis [90]. Collagen fragment is from extracellular matrix proteins depositing gradually during progression of glomerular sclerosis [97]. Glutathione-S-transferase increased where E-cadherin decreased in urine samples both associating with apoptosis and oxidative stress mechanisms in FSGS pathogenesis. Pérez, et al. performed a urinary peptide profiling to compare MCD and FSGS with each other and with healthy controls. They combined magnetic bead-based technology with MALDI-TOF/MS to find peptide profiles in urine samples of 22 patients with MCD and 22 patients with FSGS. They successfully classified about 72% of FSGS and 81% of MCD patients by a class prediction model. The peak areas corresponding to uromodulin (m/z 1913.60) and α-1-antitrypsin fragment (m/z 2392.54) showed higher and lower expressions, respectively in FSGS patients compared to MCD patients [98]. Localization of α-1-antitrypsin within sclerotic glomeruli might be related to podocytes stress and fibrogenic role of this protein in progression of FSGS [99]. Uromodulin is the most abundant protein in the urine of healthy people exclusively expressed on the epithelial cells of Henle’s loop and previous studies have proposed this protein as a marker of urinary system-related diseases [100]. Wang, et al. studied urinary proteome profile of patients with nephrotic syndrome, FSGS, MCD, and healthy controls using 2DE coupled to MS/MS. Among the most important proteins, ubiquitin-60S ribosomal protein L40 (UBA52) significantly increased in all groups of patients compared to healthy subjects, where it also increased in FSGS compared to MCD [101]. The increase in UBA52 level might reflect increased activity of the ubiquitin-proteasome system in FSGS pathogenesis. Nafar, et al. identified non-invasive diagnostic biomarkers of FSGS by urine proteomics using nanoLC-MS/MS analysis. They compared 11 FSGS patients with 6 patients with IgAN and 8 healthy subjects. Among 389 identified proteins, 77 of them were considered as potential biomarkers of FSGS. The most significantly altered proteins included CD59, CD44, IBP7, DPEP1, and Robo4 mainly involved in complement pathway, cell proliferation, activity of TRPC6, and actin cytoskeleton remodeling [102].

#### IgA nephropathy

Mucha, et al. studied urine proteome aimed at searching for novel diagnostic markers for IgAN in 30 patients and compared the results with those of 30 healthy subjects. They used Isoelectric Focusing Liquid Chromatography coupled to tandem Mass Spectrometry (IEF/LC-MS/MS) and detected the proteins> 10 KD, and they detected 18 significant differentially expressed proteins in IgAN patients. These proteins mainly belonged to complement components, coagulation factors, intracellular proteins, extracellular matrix, and trans-membrane proteins [103]. Complement activation has been shown to play a role in pathogenesis of IgA nephropathy. It has been found that, glomeruli complement system is activated by IgA via lectin or alternative pathways, leading to glomerular damage [104, 105]. Mohammadi Majd, et al. compared urine protein profile of patients with IgAN with those of healthy individuals using a combination of two multivariate statistical models including Sparse Linear Discriminant Analysis (SLDA) and Elastic Net (EN) regression model. The most significant proposed markers included up-regulation of transferrin, α-1-antitrypsin, and albumin fragments, where the most important down-regulated biomarkers were fibulin-5, prasoposin, YIP1 family member 3, and osteopontin [9]. As mentioned earlier in the study, potential role of α-1-antitrypsin was discussed in previous sections as identified in many other studies. Considering the role of fibulin-5 in tissue repair and oxidative stress-mediated renal damage, it was concluded that, decreased amounts of this protein is related to lower repair capacity of injuries mediated by oxidative stress in IgA nephropathy [106]. Prasoposin is a protein involved in sphingolipids hydrolysis, decreased levels of which might cause hyperlipidemia occurring in some types of IgAN [107]. Gene ontology results showed that, acute phase response and coagulation processes play important roles in disease progression. Krochmal, et al. investigated molecular signatures for IgAN through studying of previous transcriptomic and proteomic datasets. Among 232 proteins, 20 pathways were associated with IgAN consisting of 657 proteins for further analysis. They found 20 proteins with the highest relevance to disease pathology and finally validated 3 proteins which were the most important ones (including adenylyl cyclase-associated protein 1 [CAP1], prolylcarboxypeptidase [PRCP], and SHC-transforming protein 1 [SHC1]) by immunostaining of kidney tissue sections [108]. Gene ontology analysis showed that, a substantial part of significant proteins are involved in platelet activation processes, signaling and aggregation, which are all important in IgA pathology. In another study, Neprasova et al. identified non-invasive diagnostic biomarkers of IgAN in urine samples obtained from 11 IgAN patients and compared them with those of 19 healthy subjects and 8 patients with renal disease. They used various techniques including LC-MALDI-TOF/MS, proteins arrays, and immunomagnetic isolation of proteins. A panel of 7 biomarkers including 3 metabolites (8-hydroxy guanosine, dodecanal and leukotriene C4), 3 proteins (IgA-uromodulin complex, α-1-antitrypsin and galactose-deficient IgA1), and heparin sulfate could differentiate IgAN patients from other groups successfully [109]. Uromodulin and α-1-antitrypsin alterations were observed in previously mentioned studies. Galactose-deficient IgA1 might also be related to disease pathology and its association with renal clearance function has been observed [110]. Altered levels of tubulointerstitial heparin sulfate might be related to inflammatory responses observed in IgAN [111]. Guo, et al. discovered novel urinary protein biomarkers in IgAN patients of Uygur ethnicity compared to healthy controls using 2D-LC-MS/MS and iTRAQ analysis. Among 277 proteins, 4 proteins were validated by western blotting analysis as candidate biomarkers including TIMP1, ICAM, SERPINC1, and ADIPOQ. They showed “acute phase response signaling” as the most significant canonical pathway involved in IgAN pathogenesis [112]. Surin, et al. searched for urinary biomarkers of IgAN using 2DE coupled to MALDI-TOF-TOF/MS and western blotting analysis. They found an increase in α-1-antitrypsin, albumin fragments, and α-1-β-glycoprotein and a decrease in laminin G-like fragment (LG3) of endorepellin as the most significantly altered proteins in IgAN patients compared to healthy controls. They also showed an inverse correlation between LG3 level in IgAN and glomerular filtration rate, which was not observed in other glomerular diseases such as FSGS, Diabetic Nephropathy (DN), MGN, and lupus nephritis [113]. They hypothesized that, increased LG3 levels could inhibit angiogenesis due to its anti-angiogenic function consequently leading to loss of renal function in IgAN patients. Rocchetti, et al. performed a urine proteomics study on IgAN aimed at searching for non-invasive diagnostic and prognostic markers to monitor disease activity. They collected and analyzed urine samples obtained from 49 IgAN patients, 42 CKD patients, and 40 healthy controls by means of SELDI-TOF mass spectrometry. They identified differentially expressed proteins by a MALDI-TOF-MS followed by immunologic confirmation and validation in an independent set. Proteins with signals of 21,598 and 23,458 m/z significantly decreased in IgAN corresponding to perlecan laminin G-like peptide (LG3) and IgK light chains, respectively. They also showed that, these two proteins are inversely correlated with clinical severity features of IgAN [114]. Samavat, et al. studied on urine proteome to find diagnostic biomarkers for IgAN. The most significantly altered proteins included glycoprotein 2, vasorin, CD44, protocadherin, epidermal growth factor, CLM9, dipeptidyl peptidase IV, NHL repeat-containing protein 3, utreoglobin, and SLAM family member 5 [115]. These proteins are mainly involved in inflammatory response pathway and complement system cascade. Graterol, et al. tried to associate different histological types of lesions (according to Oxford classification) in IgAN with the urine and blood peptide profiles using a combination of magnetic bead technology and MALDI-TOF mass spectrometry. Histological characteristics included tubulointerstitial damage, endocapillary injury, and segmental glomerulosclerosis. They found 13 serum, 26 plasma, and 16 urine peptides that could differentiate IgAN patients from healthy controls. They also showed that, peptides corresponding to uromodulin, bradykinin, and α-1-antitrypsin are correlated with severity of the lesions [116]. Potential roles of uromodulin and α-1-antitrypsin in pathogenesis of glomerular diseases have been described in previous studies. Bradykinin, a peptide derived from kininogen, enhances production of prostaglandins and nitric oxide. Kinins have also been shown to have renal vasodilator and antifibrotic effects in some kidney diseases [117]. He, et al. compared two groups of IgAN patients with severe and mild pathologic presentations with each other with healthy controls. They suggested a panel of mass -to -charge ratios (m/z) as potential biomarkers of IgAN [118]. Moon, et al. investigated early stage biomarkers of IgAN and Thin-Basement Membranous Nephropathy (TBMN). They used a proteomic approach to determine protein content of urinary exosomes by means of LC-MS/MS in MSE mode. Among 1877 proteins, 4 proteins including aminopeptidase N, α-1-antitrypsin, vasorin precursor, and ceruloplasmin were selected as putative biomarkers to differentiate IgAN from TBMN [119]. Identified proteins were mostly involved in immune response, angiogenesis, coagulation, cell adhesion, and protein transport. Rocchetti, et al. studied urinary proteome profiles of IgAN patients aimed at identifying differential markers of responsiveness to Angiotensin Converting Enzyme (ACE) inhibitors therapy. They used 2DE-PAGE coupled to nano-HPLC-ESI-MS/MS and detected 3 proteins including kininogen, inter-α-trypsin-inhibitor heavy chain 4 (ITIH4), and tranthyretin as differentiating markers between responders and non-responders. Moreover, they confirmed the kininogen as a marker of responsiveness by immunoblotting method [120]. The role of ITIH4 in disease pathology is still unclear, but it might be a potential precursor for some bioactive peptides produced by plasma kallikrein. Substantial excretion of transthyretin has also been reported in a number of diseases having different levels of proteinuria [121]. Yokota, et al. investigated protein markers of IgAN in urine samples obtained from 17 patients compared to those of 10 healthy volunteers using 2D-DIGE method followed by identification of spots with LC-MS/MS. Among 172 identified proteins, α1-microglobulin showed good biomarker characteristics and was proposed as a putative marker for IgAN. Exact function of α1-microglobulin in IgAN pathology is not known but some previous studies reported increased amounts of this protein as a marker of glomerular and tubular dysfunction in renal diseases [122, 123]. The other most important identified proteins included transferrin, albumin, retinol-binding protein 4, β-globin, carbonic anhydrase I, cystatin C, and α2-glycoprotein 1 [124]. Smith, et al. investigated diagnostic markers for MGN, FSGS, and IgAN using the MALDI-MS imaging method applied to fresh-frozen renal tissues to search for molecular signatures of primary glomerulonephritis. They identified signals with 4025, 4048, and 4963 m/z as CKD development indicators. Moreover, they detected signals of 4025 and 4048 m/z as significant changes in FSGS and those of 4963 and 5072 m/z as significant changes in IgA nephropathy. Among these signals, 4048 m/z corresponded to α-1-antitrypsin proposed as a podocyte stress marker related to FSGS [125]. Kalantari, et al. identified urine prognostic markers to classify different pathologic stages of IgAN. Candidate biomarkers included afamin, leucine-rich alpha-2-glycoprotein, alpha-1-microgolbulin, ceruloplasmin, hemopexin, apolipoprotein A-I, vitamin D-binding protein, complement C3, beta-2-microglobulin, and retinol-binding protein 4. The most significant biological pathways related to these findings also included complement and coagulation pathways and the ECM-receptor interaction pathway [72]. Park, et al. identified some urinary markers in IgAN patients by 2DE method followed by MALDI-TOF mass spectrometry. Out of 216 differentially expressed spots, they finally proposed a panel of 59 protein markers for IgAN compared to healthy controls [126]. Haubitz, et al. also compared a group of IgAN patients with patients affected with other glomerular diseases including MGN, FSGS, DN, MCD, and healthy volunteers. They collected urine samples and analyzed them by means of Capillary Electrophoresis coupled with Mass Spectrometer (CE-MS). They detected a panel of polypeptides as the most significant discriminating markers for groups of patients with good sensitivity and specificity [127].

### Metabolomic studies in case of FSGS, MGN, and IgAN

Metabolomics as the closest layer to phenotype has received a great deal of attention in searching for diagnostic, prognostic, or therapeutic markers of diseases in recent years [128]. In case of CKD, several metabolomic studies have been performed mostly focusing on elucidation of markers in non-invasive collecting samples such as urine and serum. Taherkhani, et al. performed a urinary metabolomic analysis to distinguish between MGN patients and matched healthy controls using a combination of ^1^H-NMR spectroscopy and GC-MS methods. They also tried to find the most significant biomarkers by constructing a metabolic network for MGN and proposed 13 metabolites as the most important hubs in the network namely dopamine, fumarate, carnosine, nicotinamide D-ribonucleotide, pyridoxal, deoxyguanosine triphosphate, adenosine monophosphate, L-citrulline, nicotinamide, deoxyuridine, phenylalanine, tryptamine, and succinate. Gene ontology analysis identified “pyrimidine-containing compound biosynthesis process”, “purine ribonucleoside metabolic process”, and “aromatic amino acid family metabolic process” as the most significant biological processes associated with MGN pathology [8]. Kalantari, et al. studied metabolite markers related to proteinuria level as non-invasive prognostic factors in FSGS. They compared urine samples obtained from two groups of patients with proteinuria more and less than 3000 mg/day using ^1^H-NMR followed by multivariate orthogonal partial least squares-discriminant analysis and random forest statistical methods. They found 10 significantly altered metabolites as prognostic biomarkers for FSGS including citrulline, proline, dimethylamine, acetoacetate, valine, alphaketoisovaleric acid, isobutyrate, histidine, D-Palmitylcarnitine, and N-methylnicotinamide. They showed that, impairment of branched-chain amino acid degradation pathway was significantly correlated with massive proteinuria [10]. In another study, Hao, et al. investigated on urinary metabolome in 25 FSGS, 24 MGN, 14 MCD, 26 IgAN patients, and 35 healthy controls by means of ^1^H-NMR spectrometer. They revealed that, urinary levels of di- and tri-methyamine increased significantly while valine, hippurate, pyruvate, isoleucine, citrate, phenylacetylglycine, tyrosine, β-hydroxyisovalerate, and 3-methylhistidine decreased in FSGS patients compared to all other glomerulopathies [129]. Taherkhani, et al. performed a study using GC-MS/MS and ^1^H-NMR spectroscopy to identify the MGN sensitive and specific panel of metabolite biomarkers, and to reveal pathogenic pathways underlying MGN. They analyzed urine metabolome of 66 MGN patients, 31 healthy controls, and 72 disease controls (IgAN and FSGS) using advanced multivariate analyses. Receiver Operating Characteristic (ROC) curve analysis demonstrated that, the most accurate predictive model for MGN consisted of α-hydroxybutyric acid, 3,4-dihydroxymandelic acid, 5α-cholestanone, 2-hydroxyglutaric acid lactone, nicotinamide, epicoprostanol, and palmitic acid. Moreover, the following pathways were identified to be significantly involved in development of the disease: Pyrimidine metabolism (*P* = 0.000457), nicotinamide adenine dinucleotide salvage (*P* = 0.000899), transport of fatty acids (*P* = 0.00213), fatty acid biosynthesis (*P* = 0.00322), fatty acid and α-oxidation III (*P* = 0.00685), digestion of dietary lipid (P = 0.00685), stearate biosynthesis (P = 0.00685), metabolism (*P* = 0.00888), and transport of vitamins_ nucleosides and related molecules (*P* = 0.00973) [130]. In our previous study, we found that, 5 out of 9 pathways were up-regulated by 2 fatty acids: Palmitic acid and stearic acid. Palmitic acid induces Endoplasmic Reticulum (ER) stress in podocytes leading to apoptosis [131]. Thus, we hypothesized that, increased levels of urinary palmitic acid in patients with MGN, as reported by our research group, may be due to apoptotic death of podocyte cells in the glomeruli of MGN patients. We suggested that, controlling consumption of these two fatty acids might result in reducing disease progression. Gao, et al. performed parallel urine and serum GC/MS metabolomic analysis on human samples obtained from 30 patients with membranous nephropathy divided into two groups with urine protein levels higher than 3.5 g/24 h (HUPM) and lower than 3.5 g/24 h (LUPM). Comparison of LUPM and HUPM groups resulted in detection of 9 serum and 26 urine differential metabolites, most of them significantly increased in HUPM group. Their results showed that, oxidative stress and injury of kidney function were more severe in HUPM patients compared to LUPM patients [7]. Sui, et al. compared two groups of high- and low-risk IgAN patients in a metabolomic study using ^1^H-NMR spectrometry. They detected higher serum levels of phenylalanine, lactate, myo-Inositol, L6 lipids, L5 lipids,and L3 lipids as well as lower levels of α- and β-glucose, valine, phosphocholine, tyrosine, lysine, isoleucine, glycine, glycerolphosphocholine, glutamate, glutamine, alanine, acetate, 1-methylhistidine, and 3-hydroxybutyrate both in low- and high-risk patients compared to healthy controls [132]. Details on metabolomic studies are provided in Table 2.

**Table 2:** Metabolomic studies in the case of FSGS, MGN and IgAN

CKD Type	Sample Type	Method	Putative Biomarkers	Ref.
MGN	Urine	GC-MS	dopamine, fumarate, carnosine, nicotinamide D-ribonucleotide, pyridoxal, deoxyguanosine triphosphate, adenosine monophosphate, L-citrulline, nico¬tinamide, deoxyuridine, phenylalanine, tryptamine, succinate	(Taherkhani et al., 2018)
	Urine	GC-MS ^1^H-NMR	α-hydroxybutyric acid, 3,4-dihydroxymandelic acid, 5α-cholestanone, 2-hydroxyglutaric acid lactone, nicotinamide, epicoprostanol, palmitic acid	(Taherkhani et al., 2019)
	Urine, serum	GC/MS	Urine: cis-Aconitic acid, Lactose, Erythritol, Xylitol, Galactitol, Inositol, Glyceric acid, 2,4-Dihydroxybutyric acid, Threonic acid, 2-Deoxyribonic acid, 2-Ketogluconic acid, Glutaric acid, 3-Methylglutaric acid, Adipic acid, 2-Hydroxyglutaric acid, Suberic acid, 3-Hydroxysebacic acid, Mandelic acid, 4-Hydroxyphenylacetic acid, Vanillic acid, 3,4-Dihydroxybenzoic acid, 4-Hydroxyphenyllactic acid, Vanillactic acid, Cytosine, Quinolinic acid, Cholesterol Serum: m-Cresol, 2-Keto-3-methylvaleric acid, L-Asparagine, L-Serine, L-Threonine, Pyroglutamic acid, Citric acid, Glucose, Cholesterol	(Gao et al., 2012)
IgAN	Urine	LC-MALDI-TOF/MS, proteins arrays, immunomagnetic isolation	8-hydroxy guanosine, dodecanal, leukotriene C4	(Neprasova et al., 2016)
	Serum	NOESYPR and TOCSY ^1^H-NMR	phenylalanine, lactate, myo-Inositol, L6 lipids, L5 lipids and L3 lipids as well as lower levels of α- and β-glucose, valine, phosphocholine, tyrosine, lysine, isoleucine, glycine, glycerolphosphocholine, glutamate, glutamine, alanine, acetate, 1-methylhistidine and 3-hydroxybutyrate	(Sui et al., 2012)
FSGS	Urine	CPMG ^1^H-NMR	citrulline, proline, dimethylamine, acetoacetate, valine, alphaketoisovaleric acid, isobutyrate, histidine, D-Palmitylcarnitine, N-methylnicotinamide	(S. Kalantari et al., 2016)
	Urine	CPMG ^1^H-NMR	valine, hippurate, pyruvate, isoleucine, citrate, phenylacetylglycine, tyrosine, β-hydroxyisovalerate, 3-methylhistidine, di- methylamine, tri-methyamine	(Hao et al., 2013)

*MGN* Membranous glomerulonephritis, *IgAN* Immunoglobulin A nephropathy, *FSGS* Focal segmental glomerulosclerosis, *MCD* Minimal change disease

## Conclusion

In the current study, an accurate definition of CKD was provided, then it was attempted to focus on pathogenesis, molecular mechanisms, and novel therapeutic approaches related to MGN, FSGS, and IgAN. In addition, recent studies were reviewed regarding identification of potential biomarkers of each of mentioned disorders using proteomic and metabolomic technologies. Previous studies showed that, GBM thickening, podocyte injury, and deposition of Gd-IgA1-containing ICs in mesangial area of the glomeruli occur in MGN, FSGS, and IgAN, respectively. To date, renal-biopsy is gold standard diagnostic approach in nephrology, but it has some complications such as invasiveness, being dependent on pathologist’s diagnosis, and some other circumstances like renal size, infections, and hypertension. Therefore, sensitive and specific diagnostic biomarkers are needed in favor of diagnosis, prognosis, and prediction of response to therapy. Bio-fluids are valuable sources of proteins and metabolites, which have the potential to be considered as diagnostic biomarkers for CKD_s_. Moreover, urine has a higher priority in comparison with other bio-fluid samples due to several reasons; firstly, due to convenience of collection compared to other samples, especially renal tissue having limitations in collection and tissue biomarkers, which may not be applicable for all the patients. The other advantage of urine over other biofluids including serum or plasma is wide dynamic range of blood proteome causing some limitations in biomarker discovery. Moreover, urine is a source of more specific biomarkers compared to serum/plasma, as it is in direct contact with glomeruli. Thus, biomarkers identified from bio-fluids, especially urine could provide reliable and safe alternative source for diagnosis of renal diseases, besides common tissue biopsy. However, molecular biomarkers have not been used in clinical practice yet, and the next step will be to focus on identification of these biomarkers in larger sample sizes and among different nations, and to validate these markers. In addition, using robust statistical and bioinformatic tools may improve sensitivity and specificity of biomarkers. Moreover, translation of these findings from bench to clinic needs more investigations and focusing on designing of diagnostic kits and tests, which are easy to use in clinical laboratories.

## Data Availability

Data sharing is not applicable to this paper as no datasets were generated or analyzed during the current study.

## Abbreviations

^1^H-NMR: Proton-nuclear magnetic resonance
2DE: Two-Dimensional Electrophoresis
CF: Circulating Factors
CKD: Chronic Kidney Disease
CLCF1: Cardiotrophin-Like Cytokine Factor 1
DN: Diabetic Nephropathy
ESRD: End-Stage Renal Disease
FFPE: Formalin -Fixed Paraffin -Embedded
FSGS: Focal Segmental Glomerulosclerosis
GBM: Glomerular Basement Membrane
GC-MS: Gas Chromatography coupled to Mass Spectrometry
GFR: Glomerular Filtration Rate
ICs: Immune Complexes
IgAN: Immunoglobulin -A Nephropathy
iTRAQ: isobaric Tag for Relative and Absolute Quantitation
LC-MS/MS: Liquid Chromatography coupled to tandem Mass Spectrometry
MCD: Minimal Change Disease
MGN: Membranous Glomerulonephritis
MPG: Membranoproliferative Glomerulonephritis
SELDI: Surface-Enhanced Laser Desorption/Ionization
TBMN: Thin-Basement Membranous Nephropathy
TNF-α: Tumor Necrosis Factor-α
